# Quality of life and supportive care needs in prostate cancer: the impact of treatment received and care service utilization among Māori and non-Māori patients in New Zealand

**DOI:** 10.1007/s00520-025-09521-7

**Published:** 2025-05-20

**Authors:** Hui Xiao, G. David Baxter, Lizhou Liu, Tobias Hoeta, Erik Wibowo

**Affiliations:** 1 Centre for Men’s Health NZ, Centre for Health, Activity and Rehabilitation Research, School of Physiotherapy, University of Otago, PO Box 56, Dunedin, 9054 New Zealand; 2 Present Address: Exercise Oncology Lab, Faculty of Kinesiology and Physical Education, University of Toronto, Toronto, Canada; 3 Centre for Men’s Health NZ, Department of Anatomy, University of Otago, PO Box 56, Dunedin, 9054 New Zealand; 4 Present Address: School of Medical Sciences, Faculty of Medicine and Health, University of Sydney, Camperdown, Australia

**Keywords:** Prostate cancer, Care service utilization, Māori and non-Māori patients

## Abstract

**Background:**

Prostate cancer treatment can lead to significant long-term side effects that impact patients’ quality of life and supportive care needs (SCN). This study explores the associations between quality of life (QoL) and SCN among prostate cancer survivors, with a focus on the impact of treatment received, care service utilization, and the differences between Māori and non-Māori patients.

**Methods:**

Random stratified sampling data were collected from 1075 prostate cancer survivors who were diagnosed within the past 5 years. Hierarchical regression analyses examined the associations between QoL domains and SCN, adjusting for demographic, clinical, and treatment-related factors. LASSO (Least Absolute Shrinkage and Selection Operator) was used to select variables to test the interaction effects of different treatments.

**Results:**

Significant disparities were found between Māori and non-Māori patients in physical and mental health scores, care service utilization, and overall SCN. Māori men had lower scores in these areas. Most QoL domains were negatively associated with more SCN, particularly mental health and hormonal issues. Androgen deprivation therapy (ADT) exacerbates some negative effects of poor mental health and hormonal issues for non-Māori, while the use of care services and radical prostatectomy (RP) was associated with mitigating SCN for Māori patients.

**Conclusion:**

This study highlights the complex interplay between QoL, SCN, and treatment modalities among prostate cancer survivors in New Zealand. The findings underscore the need for culturally tailored supportive care services to address the unique needs of Māori patients.

**Supplementary Information:**

The online version contains supplementary material available at 10.1007/s00520-025-09521-7.

## Introduction

In New Zealand (NZ), prostate cancer is the most commonly diagnosed cancer among men, with favorable 5- and 10-year survival rates are over 95% and 90%, respectively [1]. The widespread use of prostate-specific antigen (PSA) testing has had a profound impact on prostate cancer incidence [2]. Over the past two decades, the incidence has risen from 40 cases per 100,000 in the pre-PSA era to over 100 cases per 100,000, suggesting that up to 50% of cases may be over diagnosed [2]. This places additional strain on healthcare resources by increasing the number of men requiring monitoring or treatments. Many prostate cancer survivors (PCS) suffer from long-term side effects from these treatments, such as urinary incontinence, erectile dysfunction, fatigue, depression and anxiety [3, 4]. These side effects extend far beyond the physical, deeply impacting their quality of life (QoL). Consequently, supportive care emerges as a pivotal element in enhancing QoL for PCS.

The relationship between QoL and supportive care needs (SCN) in PCS has significantly evolved as a crucial area of study over the years. Improving QoL and addressing unmet needs go beyond known physical, psychological and informational aspects. The variations in treatments and outcomes also affect the post-treatment lives of PCS. Research has demonstrated that while medical treatments for prostate cancer can extend life expectancy, they often come with side effects that can severely impact the QoL of survivors [5]. Patients who received external radiation and brachytherapy tend to have a worse bowel function, but better or equivalent urinary and sexual function compared with those who had prostatectomy [6]. A comprehensive systematic review highlighted that a significant proportion of PCS continues to experience substantial unmet SCN after treatments, particularly in areas concerning sexual health, mental wellbeing, and coping strategies [7]. A study across seven European countries also showed that factors such as the stage of cancer at diagnosis, treatment modality received (e.g., surgery, radiotherapy, hormonal therapy), and socio-demographic characteristics (age, income level, and educational background) play a pivotal role in shaping these needs [8].

In NZ, the utilization of supportive care services is fragmented and inequitable. The supportive care services for PCS in NZ are predominantly provided in urology and oncology services at Te Whatu Ora—Health NZ. Although the government raised the investment by committing approximately $4.2 million annually to the Cancer Psychological and Social Support Initiative [9] and there are community support organizations consistently providing care services including peer support groups, exercise programs, education seminars/webinars, and online support consultations. PCS in NZ still frequently report unmet needs after completing active treatments, which are not adequately addressed by the current supportive care services [10]. Māori, the Indigenous people of Aotearoa NZ, experience higher SCN yet utilize fewer services due to structural inequities and unique cultural barriers [3, 4]. Additionally, many Pākehā (NZ European) men receive treatment in the private sector rather than through Te Whatu Ora—Health NZ [3]. While private care often provides advantages such as shorter wait times and greater flexibility in choosing treatment options, it may limit access to integrated supportive care services, which are more systematically embedded within the public healthcare system. In contrast, Māori men face distinct challenges, including barriers to accessing follow-up care and limited knowledge about prostate cancer symptoms [11]. These factors are compounded by cultural differences in healthcare preferences, with Māori patients often requiring tailored supportive care services that address their unique cultural and social needs [12]. These disparities emphasize the need for culturally tailored interventions to meet diverse patient needs and improve QoL to ensure equitable care for all men with prostate cancer in NZ.

Therefore, the primary aim of this study is to explore the associations between QoL and SCN among PCS and assess how different treatment modalities and supportive care service utilization moderate these relationships. This allows us to dissect the nuances of treatment effects, potentially identifying which modalities are associated with better or worse QoL and higher or lower needs for supportive care. Understanding these relationships will inform targeted supportive care strategies, ultimately enhancing QoL and addressing healthcare disparities for all PCS in NZ.

## Method

### Participants and procedure

This study was conducted in NZ in October 2023. Data for 17,886 men diagnosed with prostate cancer within the past 5 years were obtained from the electronic database of the Cancer Registry, Ministry of Health, and cross-verified with the Prostate Cancer Outcome Registry New Zealand. Only non-metastatic PCS with Gleason Scores ≤ 7 and TNM staging ≤ 2 was included, as metastatic PCS often have distinct clinical trajectories and SCN [13]. Participants were randomly stratified into four groups (*N* = 1000 each) by diagnosis dates (within 1 year, 1 to 3 years, 3 to 5 years) and ethnicity, with an additional group exclusively composing of Māori men equally distributed across these timeframes. The total sample size for the study was 4000 participants.

Participant engagement (Appendix A) included consultations with a Māori co-investigator and prostate cancer supportive care providers, and a pilot study was conducted using face-to-face and online surveys. A study webpage was created providing detailed information, including informed consent and confidentiality assurances [14]. Custom postcards (Appendix B) with links and QR codes were distributed, enabling online survey completion via REDCap or requests for paper-based questionnaires. This study was approved by the New Zealand Health and Disability Ethics Committee (HDEC) (ref_2022 EXP 11640) and conducted in accordance with the Declaration of Helsinki. Māori Research Consultation was made through the Ngāi Tahu Research Consultation Committee, University of Otago (ref: 5767_21979).

### Survey measurements

The development of the survey instruments and measurement approach has been previously reported [4]. Table 1 provides an overview of the key instruments and measures utilized in this study. SCN were assessed using the Supportive Care Needs Survey Short Form (SCNS-34SF [15]. For Māori participants, a culturally adapted version was developed based on the Supportive Care Needs Assessment Tool for Indigenous People (SCNAT-IP). The SCNAT-IP was originally developed for Indigenous Australian populations, and we adapted it for Māori participants in consultation with a Māori co-investigator to ensure cultural relevance [16]. QoL was evaluated through Expanded Prostate Cancer Index Composite Short Form EPIC-26, which assesses prostate cancer-specific symptoms, and Medical Outcomes Study SF-12v2 (SF-12v2), which measures physical and mental health domains [17]. The Care Service Utilization Scale was developed drawing on three principal sources: [1] the methodological framework established by Cockle-Hearne et al. [8], which utilized expert input from clinical specialists, [2] evidence synthesized from a systematic review by King et al. [18], and (3) consultations with key representatives from the Prostate Cancer Foundation NZ and Cancer Society NZ [8, 18] (Appendix A). Additionally, socio-demographic and medical data were collected to account for potential confounding factors in the analysis.

**Table 1:** Summary of measurements used in the study

Domain	Instrument/source	Description	Scoring
Supportive care needs	Supportive Care Needs Survey Short Form (SCNS-34SF)	Assesses 34 supportive care needs across multiple domains. A Māori-adapted version incorporating cultural elements was used for Māori participants	Standardized score (0–100), higher scores indicate greater needs
QoL and treatment	Expanded Prostate Cancer Index Composite Short Form (EPIC-26) and SF-12v2	EPIC-26: Assesses urinary, bowel, sexual, and hormonal symptoms; SF-12v2: Assesses physical and mental health	Standardized score (0–100), higher scores indicate better QoL
Care service utilization	Self-developed scale based on existing frameworks and stakeholder input	Measures advice and support received from clinical professionals, NGOs, and other sources (19 items)	Sum of total scores (0–100), higher scores indicate greater utilization of services
Control variables	Socio-demographic and medical data	Includes relationship status, education, income, employment, smoking, and medical conditions	Categorical variables; comparisons made with a reference category

Inter-scale correlation between EPIC26 and SF-12 has indicated that efficient QOL assessment can be achieved by co-administering EPIC26 with SF-12

The Cronbach’s alpha for SCNAT-IP and supportive care utilization scale was 0.98 and 0.83 respectively, indicating a high level of internal consistency and suggesting that the items reliably measure a cohesive underlying construct

### Data analysis

The analysis was conducted using Stata version 17.0. Observations with over 80% missingness on any scale or an entirely missing instrument were excluded. Mean imputation was used after observing data patterns as missing completely at random. Descriptive statistics were computed to describe the participants’ socio-demographic characteristics, QoL and SCN. The relationship between QoL and SCN was analyzed through ordinary least squares (OLS) regression. Multivariate models were employed, adjusting for different control variables to assess their impact. The basic model explored the direct association between QoL and SCN using a single regression model with all QoL domains included. The adjusted model incorporated control variables, while the interaction model included treatment and service utilization variables based on the adjusted model. Due to the high number of interaction terms tested, the Least Absolute Shrinkage and Selection Operator (LASSO) linear regressions were employed for the interaction model to handle the potential multicollinearity and to perform variable selection. Similar hierarchical regression models were used for Māori participants using the sum score of SCNAT-IP Māori version as the outcome variable. Statistical significance was determined at a *p*-value of less than 0.05. Beta (*β*) coefficients and 95% confidence intervals (CIs) were reported for each model.

## Results

**Fig. 1:**
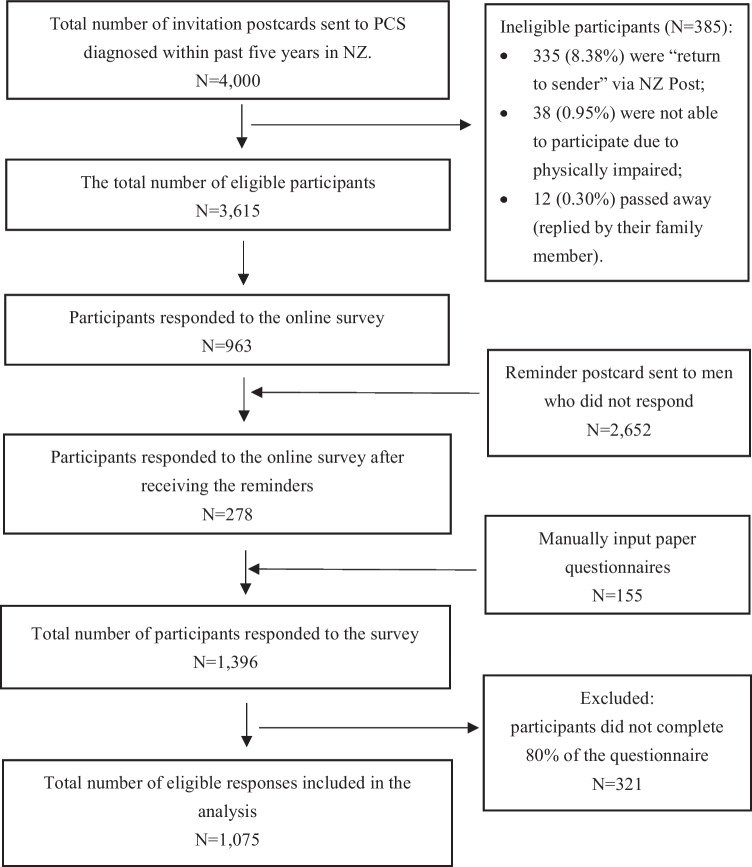
Flow diagram of patient selection for the data analyses

Data was based on 1075 complete questionnaires (Fig. 1). Participants’ demographics are in Table 2, with NZ European men making up 74.60% and Māori men 16.19% due to oversampling. The average age of participants was 67.21 ± 15.62 years, with a similar age between the two ethnic groups. Most participants were married (71.04%) and lived with spouses (77.11%). Māori men were more likely to be widowed or divorced (38.04% vs. 14.03%). The retirement rate of 67.07% aligned with the age distribution of the cohort. Educationally, 48.70% had a high school diploma and 42.50% had a university education, with 47.13% of non-Māori participants reporting university or postgraduate education compared to 16.85% of Māori men. Economically, 45.27% of participants reported a household income between $30 k and $100 k, with comparable rates among Māori (42.39%) and non-Māori (46.07%). However, non-Māori participants were more likely to report household incomes exceeding $100 k (28.14%) compared to Māori participants (14.13%). Smoking prevalence was 8.73%, with significantly higher rates among Māori men (34.79%) compared to non-Māori men (3.52%). Approximately 48.83% reported no chronic medical conditions. Common health issues included diabetes (13.77%), heart-related ailments (13.76%), circulation problems (14.51%), asthma or emphysema (15.81%), and stomach ulcers (10.42%).

**Table 2:** Sociodemographic characteristics of study participants stratified by ethnicity (Māori vs non-Māori)

	Total (%) (*N*=1075)	Māori men (%) (*N* = 184)	Non-Māori (%) (*N* = 891)
Age	67.21 ± 15.62	66.54 ± 13.81	67.49 ± 16.66
Ethnicities			
NZ European	802 (74.60)		
Māori	174 (16.19)		
Cook Islands Māori	10 (0.93)		
Samoan	9 (0.84)		
Chinese	8 (0.74)		
Indian	16 (1.49)		
Niuean	7 (0.65)		
Others	49 (4.56)		
Relationship			
Living with spouse	829 (77.11)	95 (51.63)	734 (82.45)
In a relationship, but not living together	54 (5.02)	16 (8.70)	38 (4.24)
Not in a relationship	192 (17.87)	73 (39.67)	119 (13.32)
Education			
Below high school level	68 (6.30)	33 (17.93)	35(3.92)
High school level	524 (48.70)	109 (59.24)	415 (46.54)
University level	274 (25.43)	21 (11.41)	253 (28.37)
Postgraduate level	183 (17.07)	14 (7.61)	169 (18.95)
Prefer not to say	26 (2.50)	7 (3.80)	19 (2.22)
Marital status			
Married	765 (71.04)	83 (45.11)	682 (73.95)
Never married	57 (5.35)	16 (8.70)	41(2.52)
Separated	33 (3.03)	15 (8.15)	18 (2.24)
Divorced	135 (12.61)	45 (24.46)	90 (7.84)
Widower	85 (7.97)	25 (13.59)	60 (6.44)
Household income			
Less than $10 k	29 (2.72)	24 (13.04)	5 (0.52)
10 k–30 k	176 (16.32)	46 (25.00)	130 (14.53)
30 k–100 k	488 (45.27)	78 (42.39)	410 (46.07)
More than 100 k	277 (25.90)	26 (14.13)	251 (28.14)
Prefer not to say	105 (9.79)	9 (4.89)	96 (10.73)
Employment			
Full-time	228 (21.22)	30 (16.30)	198 (22.22)
Part-time	102 (9.51)	17(9.24)	85 (9.54)
Seeking opportunities	12 (1.10)	4 (2.17)	8 (0.85)
Retired	721 (67.07)	128 (69.57)	593 (65.55)
Prefer not to say	12 (1.10)	5 (2.72)	7 (0.85)
Smoke status			
No	980 (91.16)	120 (65.21)	860 (96.48)
Yes	95 (8.73)	64 (34.79)	31 (3.52)
Treatment			
Expectant management watchful waiting	201 (18.70)	41 (23.56)	160 (17.76)
Radical prostatectomy	461 (42.88)	72 (41.38)	389 (43.17)
External Beam Radiation	299 (27.81)	50 (28.74)	249 (27.64)
Radioactive Seed implantation	72 (6.70)	22 (12.64)	50 (5.55)
Orchiectomy	21 (1.95)	3 (1.72)	18 (2.00)
Androgen deprivation therapy	184 (17.12)	45 (25.86)	139 (15.43)
Hormone pills	57 (5.30)	14 (8.05)	43 (4.77)
Others	85 (7.91)	21 (11.41)	64 (7.18)
Medical conditions			
No chronic medical	525 (48.83)	56 (30.43)	469 (52.64)
Diabetes	148 (13.77)	67 (36.41)	81 (9.09)
Heart attack, chest pain	147 (13.76)	27 (14.67)	120 (13.47)
Stroke	56 (5.21)	26 (14.13)	30 (3.37)
Amputation	28 (2.60)	9 (4.89)	19 (2.13)
Circulation problems	156 (14.51)	49 (26.63)	107 (12.01)
Asthma or emphysema	170 (15.81)	40 (21.74)	130 (14.59)
Stomach ulcer, irritable bowel	112 (10.42)	30 (16.30)	82 (9.20)
Kidney disease	57 (5.30)	16 (8.70)	41 (4.60)
Major depression	59 (5.49)	22 (11.96)	37 (4.15)
Seizures	18 (1.67)	2 (1.09)	16 (1.80)
Alcoholism or alcohol problem	57 (5.30)	28 (15.22)	29 (3.25)
Drug problems	35 (3.26)	18 (9.78)	17 (1.91)

Table 3 shows the disparities in QoL and SCN outcomes between Māori and non-Māori. Specifically, physical and mental health scores for Māori were significantly lower than those for non-Māori (physical health: *d* = − 0.32, *p* < 0.001; mental health: *d* = − 0.92, *p* < 0.001). EPIC-26 scores across domains such as urinary incontinence, bowel, and sexual functioning indicated greater adverse impacts on Māori (e.g., urinary incontinence: *d* = − 0.48, *p* < 0.001; bowel: *d* = − 0.82, *p* < 0.001). Furthermore, care service utilization scores also demonstrated a significantly lower care utilization among Māori (26.20) compared to non-Māori (41.84) (*d* = − 0.79, *p* < 0.001). Correspondingly, Māori men had significantly higher overall standardized SCN scores (*d* = 0.97, *p* < 0.001) compared to non-Māori.

**Table 3:** Comparison of scores for QoL, service utilization, and supportive care needs between Māori and non-Māori participants

	Total (*N* = 1075)	Māori	Non-Māori	*d*	*P* value
SF12v2					
Physical health	44.39 (7.27)	42.47 (7.20)	44.78 (7.23)	− 0.32	< 0.001
Mental health	52.08 (9.84)	44.96 (11.48)	53.54 (8.80)	− 0.92	< 0.001
EPIC-26					
Urinary incontinence	76.16 (20.89)	67.87 (20.80)	77.76 (20.53)	− 0.48	< 0.001
Urinary obstructive	83.57 (16.78)	69.43 (23.79)	86.30 (13.46)	− 1.08	< 0.001
Bowel	86.75 (17.01)	75.58 (23.98)	88.90 (14.35)	− 0.82	< 0.001
Sexual	30.43 (24.57)	21.77 (21.59)	32.10 (24.77)	− 0.43	< 0.001
Hormonal	83.95 (16.35)	73.43 (22.35)	85.99 (14.05)	− 0.80	< 0.001
Care service utilizations	39.05 (20.71)	26.20 (21.81)	41.84 (19.38)	− 0.79	< 0.001
SCNS					
Overall needs		41.89 (31.44)	21.22 (33.01)	0.97	< 0.001

Comparison of independent and dependent variables between Māori men and non-Māori men

^Æ^Two-sample *T*-tests: mean (standard deviation, SD) was reported

All data as standardized from 0 to 100

Tables 4 and 5 present the regression analyses for non-Māori and Māori participants, respectively. For non-Māori patients, poorer mental health was the strongest predictor of higher SCN in the basic model (*β* = –8.50, *p* < 0.001), and several symptom-specific QoL issues (e.g., urinary and bowel problems) were also associated with greater needs in unadjusted analyses. After adjusting for covariates, mental health remained a dominant predictor of SCN (*β* = –5.09, *p* = 0.003), and physical health became a significant predictor as well (*β* = –4.23, *p* = 0.014). In the interaction model, ADT use further exacerbated the impact of poor mental health on SCN (β_interaction = –4.27, *p* = 0.003). Additionally, PCS with bowel dysfunction had especially high SCN if they were on ADT (β_interaction = –2.80, *p* = 0.047) or managed with watchful waiting (β_interaction = –4.04, *p* = 0.008).

**Table 4:** Hierarchical regression models for QoL and supportive care needs (non-Māori)

Overall supportive care needs (non-Māori)	Basic model				Adjusted model				Interaction model			
	*β*	*P*	95% CI		*β*	*P*	95% CI		*β*	*P*	95% CI	
Physical health	0.31	0.483	− 0.56	1.17	− 0.11	0.831	− 1.10	0.13	− 4.16	0.022	− 7.71	− 0.62
Mental health	− 3.95	< 0.001	− 4.99	− 2.91	− 3.49	< 0.001	− 4.73	− 0.32	− 2.47	0.098	− 5.40	0.46
Urinary incontinence	− 0.83	0.069	− 1.72	0.07	− 0.90	0.085	− 1.93	0.01				
Urinary obstructive	− 0.77	0.179	− 1.89	0.35	− 2.22	0.002	− 3.62	0.01				
Bowel	− 2.26	< 0.001	− 3.29	− 1.23	− 2.37	< 0.001	− 3.58	− 0.06	− 3.78	0.009	− 6.62	− 0.93
Sexual	− 1.38	0.002	− 2.23	− 0.52	− 1.36	0.005	− 2.31	− 0.02	− 1.95	0.077	− 4.12	0.21
Hormonal	− 3.86	< 0.001	− 4.96	− 2.76	− 5.41	< 0.001	− 6.71	− 0.17	− 5.81	< 0.001	− 7.26	− 4.36
Relationship (vs living with spouse)												
In a relationship, not living together					− 0.27	0.924	− 5.73	5.11				
Not in a relationship					1.08	0.621	− 3.19	8.71				
Marital status (vs never married)												
Married					2.75	0.275	− 2.19	9.72				
Separated					0.01	0.998	− 7.27	2.77				
Divorced					1.58	0.558	− 3.72	10.89				
Widower					− 0.29	0.922	− 6.05	1.61	− 3.07	0.072	− 6.41	0.27
Employment (vs full-time)												
Part-time					− 2.70	0.128	− 6.19	1.41				
Seeking opportunities					3.43	0.497	− 6.46	9.42				
Retired					− 0.70	0.597	− 3.31	1.93				
Prefer not to say					13.11	0.118	− 3.32	23.19	6.01	0.208	− 3.34	15.36
Smoke status (vs non-smoker)												
Smoke					− 1.25	0.609	− 6.03	3.53				
Education (vs below high school level)												
High school level					− 5.51	0.036	− 10.65	− 0.38	1.25	0.579	− 3.19	5.70
University level					− 5.05	0.062	− 10.36	0.26				
Postgraduate level					− 6.19	0.029	− 11.75	− 0.62				
Prefer not to say					− 5.17	0.312	− 15.21	4.87				
Household income (vs below 100 k)												
More than 100 k					− 0.40	0.744	− 2.82	2.02				
Medical conditions (vs no conditions)												
Diabetes					− 1.10	0.481	− 4.15	1.95				
Heart attack, chest pain					− 0.83	0.526	− 3.39	1.73				
Stroke					− 3.12	0.179	− 7.66	1.43				
Amputation					1.55	0.609	− 4.39	7.48				
Circulation problems					2.04	0.145	− 0.70	4.79	− 2.00	0.122	− 4.54	0.53
Asthma or emphysema					1.55	0.217	− 0.91	4.01				
Stomach ulcer, irritable bowel					0.21	0.893	− 2.83	3.24				
Kidney disease					3.39	0.109	− 0.76	7.53	− 2.89	0.141	− 6.75	0.96
Major depression					− 0.43	0.852	− 4.97	4.10				
Seizures					− 5.08	0.121	− 11.50	1.33				
Alcoholism or alcohol problem					− 3.68	0.178	− 9.04	1.67				
Drug problems					15.10	< 0.001	7.64	22.55	− 10.23	0.001	− 16.47	− 4.00
Significant interactions									β_int	*P*	95% CI	
With mental health												
ADT									− 4.27	0.003	− 7.11	− 1.42
With bowel												
Watchful waiting									− 4.04	0.008	− 7.04	1.05
ADT									− 2.80	0.047	− 5.67	−0.08

**Table 5:** Hierarchical regression models for QoL and supportive care needs (Māori)

Overall supportive care needs (Māori)	Basic model				Adjusted model				Interaction model			
	*β*	*P*	95% CI		*β*	*P*	95% CI		*β*	*P*	95% CI	
Physical health	− 2.21	0.121	− 5.00	0.59	− 4.23	0.014	− 7.59	− 0.87				
Mental health	− 8.50	< 0.001	− 11.37	− 5.63	− 5.09	0.003	− 8.38	− 1.80	− 6.30	< 0.001	− 9.21	− 3.40
Urinary incontinence	− 3.07	0.057	− 6.24	0.10	− 5.53	0.001	− 8.80	− 2.26				
Urinary obstructive	− 4.60	0.006	− 7.83	− 1.36	− 0.22	0.907	− 3.97	3.53				
Bowel	− 4.11	0.007	− 7.08	− 1.15	− 0.04	0.985	− 3.62	3.55				
Sexual	0.63	0.719	− 2.84	4.10	0.22	0.886	− 2.79	3.22				
Hormonal	− 4.68	< 0.001	− 7.35	− 2.12	− 8.69	< 0.001	− 11.75	− 5.63	− 8.21	< 0.001	− 10.76	− 5.66
Relationship (vs living with spouse)												
In a relationship, not living together					11.12	0.145	− 3.92	26.15				
Not in a relationship					13.11	0.066	− 0.87	27.10				
Marital status (vs never married)												
Married					− 8.99	0.621	− 5.55	32.44	2.31	0.072	− 0.21	4.83
Separated					6.66	0.386	− 8.54	21.86				
Divorced					17.35	0.004	5.60	29.10	5.94	0.048	0.04	11.84
Widower					8.90	0.172	− 3.96	21.77				
Employment (vs full-time)												
Part-time					− 10.67	0.026	− 20.01	− 1.33				
Seeking opportunities					17.66	0.072	− 1.60	36.93	13.95	0.019	2.31	25.58
Retired					− 3.68	0.404	− 12.41	5.05				
Prefer not to say					5.05	0.773	− 29.72	39.83				
Smoke status (vs non-smoker)												
Smoke					22.27	< 0.001	12.35	32.19	18.13	< 0.001	23.48	12.77
Education (vs below high school level)												
High school level					6.17	0.293	− 5.42	17.75				
University level					12.78	0.062	− 0.67	26.22				
Postgraduate level					13.58	0.059	− 0.55	27.72				
Prefer not to say					− 10.24	0.290	− 29.39	8.90				
Household income (vs below 100 k)												
More than 100 k					− 5.27	0.245	− 14.22	3.68				
Medical conditions (vs no conditions)												
Diabetes					− 2.87	0.520	− 11.72	5.98				
Heart attack, chest pain					6.35	0.102	− 1.29	13.99	8.39	0.003	13.93	2.85
Stroke					− 1.39	0.765	− 10.63	7.84				
Amputation					20.75	0.005	6.53	34.97				
Circulation problems					2.96	0.478	− 5.30	11.22				
Asthma or emphysema					− 0.84	0.864	− 10.54	8.86				
Stomach ulcer, irritable bowel					− 7.37	0.114	− 16.54	1.80				
Kidney disease					− 0.85	0.859	− 10.27	8.58				
Major depression					− 2.89	0.633	− 14.88	9.10				
Seizures					omitted							
Alcoholism or alcohol problem					7.21	0.196	− 3.79	18.20				
Drug problems					− 1.38	0.824	− 13.68	10.92				
Significant interactions									β_int	*P*	95% CI	
With physical health												
Service utilization									0.29	0.046	0.01	0.58
With mental health												
Radical prostatectomy									6.36	0.002	2.32	10.41
Service utilization									0.14	0.033	0.01	0.26
With hormonal												
Radical prostatectomy									3.69	0.028	1.12	7.52

LASSO inference was applied in the interaction model to effectively address high collinearity and perform variable selection. Only the variables selected by LASSO were included in the interaction model

To ensure that the interaction effects are built upon robust main effects, significant interactions are presented where the corresponding relationships in the adjusted model were also significant

For Māori PCS, poorer mental health (*β* = –8.50, *p* < 0.001) and more severe hormonal symptoms (*β* = –4.68, *p* < 0.001) were significantly associated with higher SCN in the basic model, alongside urinary obstructive and bowel issues. In the adjusted model for Māori, mental health (*β* = –5.09, *p* = 0.003) and hormonal issues (*β* = –8.69, *p* < 0.001) remained significant, and physical health emerged as a significant predictor of SCN (*β* = –4.23, *p* = 0.014). In the interaction model for Māori, greater utilization of supportive care services buffered some of the negative effects of poor QoL on SCN: the association of poor physical health with SCN was slightly reduced with higher service use (β_interaction = 0.29, *p* = 0.046), as was the association of poor mental health with SCN (β_interaction = 0.14, *p* = 0.033). Moreover, having undergone RP mitigated some of the needs associated with poor mental health (β_interaction = 6.36, *p* = 0.002) and hormonal issues (β_interaction = 3.69, p = 0.028) in Māori patients.

## Discussion

This study examined the associations between QoL and SCN in PCS, highlighting significant disparities between Māori and non-Māori patients in NZ. Most QoL domains were negatively associated with SCN, particularly mental health and hormonal issues remaining significant in both groups. Our findings also reveal several important insights into how different treatment modalities and the utilization of care services interact with QoL domains to influence SCN. It is shown that ADT and watchful waiting appeared to exacerbate some of the negative effects, whereas the RP and use of the care services in Māori groups may mitigate these effects.

Māori men demonstrated markedly lower scores in both the physical, and mental health scores, aligning with known disparities linked to social characteristics, biological differences, variations in treatment received, and disparities in access to or utilization of supportive care services [12, 17, 19]. For instance, our data reveal that Māori men are more likely to have below high school-level education, live alone or be in non-cohabiting relationships, and report lower household incomes. These align with earlier studies, which emphasize how socioeconomic disadvantages and social isolation contribute to poorer mental health outcomes and lower QoL in Māori [20]. Notably, our adjusted model shows that physical health significantly affects SCN among Māori patients, whereas it remains insignificant for non-Māori patients. This discrepancy may be due to the higher prevalence of comorbidities among Māori PCS, which increase the physical health burden [12]. Interestingly, after incorporating all the treatment interaction terms, the relationship between physical health and SCN becomes significant for non-Māori patients in the interaction model. This suggests that the impact of treatments on SCN is also substantial for non-Māori patients. There are consistent themes regarding the influence of mental health and hormonal issues on SCN. Earlier research highlighted that PCS with psychological distress had greater unmet SCN across all domains [21, 22]. Our study corroborates these findings, demonstrating that poor mental health significantly increases SCN. Moreover, hormonal issues also emerged as a critical factor in both groups, aligning with findings highlighting the profound impact of treatment-related hormonal changes on QoL [23].

Our results indicate that treatment modalities significantly influence SCN. Māori men exhibited higher rates of watchful waiting, ADT, and hormonal therapy but fewer radical prostatectomies compared to non-Māori, consistent with previous research in NZ [23]. The previous study also shows that RP is associated with an increased risk of urinary incontinence, and ADT is associated with erectile dysfunction and urinary incontinence [24]. ADT notably exacerbated negative associations between mental health, bowel function, and SCN, possibly due to hormonal disruptions increased rates of anxiety and depression, as well as the incidence of inflammatory bowel disease among PCS [25–28]. Symptoms such as fatigue, loss of muscle mass, cognitive decline, and sexual dysfunction further contribute to overall psychological distress [29, 30]. This added burden can worsen the mental health of patients, thereby increasing their SCN.

For bowel issues, the exacerbation may result from ADT negatively altering gut microbiota and gastrointestinal function [31]. Watchful waiting as a treatment involves monitoring the cancer without active intervention until symptoms worsen. This can lead to a deterioration in bowel function over time and an increase in SCN. Our interaction analysis revealed that watchful waiting significantly strengthened the negative association between bowel function and SCN among non-Māori PCS. While previous studies typically linked bowel dysfunction to active treatments like surgery or radiotherapy [32]. Our results suggest that watchful waiting, while less invasive, may still contribute to bowel-related challenges. This may reflect disease progression, delayed symptom management, or prolonged uncertainty and anxiety, potentially exacerbating psychological distress, creating a cycle of increasing SCN. In contrast, RP appear to mitigate these negative associations of SCN with mental health and hormonal issues for Māori patients. It is possibly due to the definitive nature of surgery, providing immediate cancer removal and reducing psychological distress associated with disease management. While Māori men in our study were more likely to receive less invasive treatments, those who underwent RP experienced notable benefits in mitigating SCN. However, the role of RP in older men with low-risk prostate cancer remains uncertain, as previous studies have found no significant survival benefit in this population [33, 34]. Conversely, RP has shown benefits for patients with intermediate- and high-risk disease, potentially preventing metastatic seeding of cancer cells and improving disease control [35].

It is also important to recognize that “treatment received” does not always equate to “treatment preference”. In many cases, systemic barriers and healthcare access constraints frequently influence treatment decisions, especially among Māori men [3]. This context is crucial when interpreting the apparent benefits of RP on SCN: while some patients clearly benefit from surgical intervention, not all have equal access to this option. Recognizing these nuances underscores the importance of care service utilization in further mitigating SCN. Our interaction model reveals that supportive care service utilization significantly reduces SCN related to physical and mental health among Māori patients. Although the interaction coefficients are relatively small, this positive outcome may reflect improvements in the accessibility and cultural sensitivity of care services in NZ. Given that Te Ao Māori emphasizes the interconnectedness between people, community, and environment, culturally sensitive healthcare services are crucial in shaping how health and care services are perceived and utilized by Māori [36, 37]. This is also reflected in our demographic data where proportionally more Māori patients receive non-invasive or minimally invasive treatments than NZ European patients, but fewer Māori men are using care services. It is, therefore, crucial to enhance these services to ensure they meet the cultural and health needs of Māori patients effectively.

Finally, our results do not imply the inherent superiority of any treatment modality. Instead, treatment interactions indicate that ADT might increase SCN among those with poor mental health or bowel function but could benefit those with fewer issues. Similarly, RP should be considered thoughtfully, especially for Māori patients with good mental health and fewer hormonal issues, acknowledging that surgery itself can also lead to increased SCN. Patient-centered decision-making and tailored supportive interventions are therefore essential to minimize treatment-related adverse effects.

### Strength, limitation, and future implications

A key strength of this study is the deliberate oversampling of Māori participants, which increased their representation to 16.9% of the sample (versus ~ 9.4% in the registry) and improved the reliability of cross-ethnic comparisons. A notable methodological consideration in this study is the use of two versions of the SCN assessment (SCNS-34SF for non-Māori and an adapted SCNAT-IP for Māori), enabling us to capture both general and culturally specific needs. However, this also means that direct comparisons between Māori and non-Māori groups should be interpreted with caution, as differences may reflect both true disparities in unmet needs and measurement variances arising from the distinct survey instruments. Despite this oversampling, the relatively small sample size of Māori PCS still presented challenges for the analysis. This constrained sample size necessitated the removal of some high collinearity variables to maintain the robustness and interpretability of the statistical models, consequently reducing the statistical power of the interaction analyses. Additionally, while the study adjusted for various demographic and clinical factors, there might be unmeasured confounding variables influencing the outcomes, such as residential locations affecting access to healthcare. The limited representation of specific minority groups, such as Sāmoan and Niuean PCS, required their inclusion in the broader non-Māori group, potentially obscuring their unique SCN. One other significant limitation is the cross-sectional design, which restricts the ability to infer causality between the variables studied.

Despite these limitations, the findings from this study have important implications for future research and clinical practice. The significant disparities in SCN and QoL between Māori and non-Māori PCS underscore the necessity for culturally tailored supportive care interventions. Incorporating principles from Te Ao Māori into care services could ensure culturally relevant and holistic care, potentially mitigating the inequities observed [38]. Future research should focus on these identified interacting treatments and apply advanced analytical techniques, such as Latent Profile Analysis [39], to identify distinct subgroups of patients with similar SCN. Longitudinal studies following patients over time (before, during, and after treatment) are warranted to see how QoL and SCN evolve throughout the cancer journey. Such research would provide deeper insights into the timing and impact of different treatments on QoL and needs, and would help in designing more effective, personalized supportive care strategies for PCS.

## Conclusion

This study explored the complex associations between QoL and SCN among PCS in NZ, highlighting significant disparities between Māori and non-Māori patients. We found negative associations between most QoL domains and SCN, especially concerning mental health and hormonal issues. Our study is the first in NZ to explore the interaction effects of different treatments on SCN and QoL. Among these, ADT can exacerbate some negative effects, while care service use might mitigate physical and mental health impacts for Māori. These findings highlight the need for tailored supportive care services, improving treatment choices, and clinical outcomes, and reducing healthcare disparities, particularly for underrepresented groups like Māori men.

## Supplementary Information

Below is the link to the electronic supplementary material.

Supplementary Material 1 (DOCX 37.7 KB)

Supplementary Material 2 (PDF 904 KB)

## Data Availability

No datasets were generated or analysed during the current study.
