# Supplementary material for: Quality of life and supportive care needs in prostate cancer: the impact of treatment received and care service utilization among Māori and non-Māori patients in New Zealand
Source: Support Care Cancer. 2025 May 20;33(6):483. doi: 10.1007/s00520-025-09521-7 (PMC12092496; doi:10.1007/s00520-025-09521-7)
Supplement: Supplementary file 1 — Supplementary Material 1 (DOCX 37.7 KB) [file 520_2025_9521_MOESM1_ESM.docx]

# Participant engagement

The following strategies were employed to engage PCS in the study:

(a) Consulting: Expert consultations were held with a Māori co-investigator and community prostate cancer supportive care providers.

(b) Involving: A pilot study was conducted using both face-to-face and online surveys with PCS recruited from the Prostate Cancer Foundation of New Zealand. All inquiries, reservations, or errors were documented. A feedback form was also distributed that featured a rating scale from 1 to 4, assessing relevance, clarity, and simplicity. Overall feedback was also collected. The survey measurements are listed in the Supplementary Documents.

(c) Informing: A study webpage was created to provide detailed study information, including an introduction, survey access links, information sheets, consent forms, and summaries of related work. All participants provided informed consent prior to participating in the study. Participants were assured of the confidentiality of their responses and their right to withdraw from the study at any time without penalty.

(d) Implementing: Custom survey invitation postcards, featuring a link to the study webpage and a QR code for the online survey, were distributed to participants. These postcards provided participants with the option to either complete the survey online via the REDCap (Research Electronic Data Capture) database or request paper-based questionnaires by contacting the research team.

# Measurements

## Supportive care needs

In our study, we employed the Supportive Care Needs Survey Short Form (SCNS-34SF) to evaluate the diverse care requirements of non-Māori participants (Boyes et al., 2009). This 34-item instrument has been extensively utilized in various international studies focusing on the supportive care needs of prostate cancer patients (Cockle-Hearne et al., 2013; Ream et al., 2008). Since Māori men may have different needs, we also developed a Supportive Care Needs Survey Māori version based on the revision of supportive Care Needs Assessment Tool for Indigenous People (SCNAT-IP) for Māori participants (Garvey et al., 2012). With advice, and without changing the nature of the questions and options, we incorporated elements related to Māori culture into the questionnaires. For example, for the question “In general, would you say your health is? Excellent, very good, good, fair, poor”, apart from the explanation of health meaning physical and mental health, spiritual and whānau (family) health were also added as examples (Many Māori place their spiritual health and whānau (family) as integral parts of their general health (Hoeta et al., 2020). Additionally, a seven-item prostate-specific module is added to both scales that measure needs relating to urinary (2 items) and bowel function (1 item), masculine self-image (4 items). The overall needs are the sum score of each domain and standardized from 0-100 with higher score indicating high needs

## QoL and treatments

The Expanded Prostate Cancer Index Composite Short Form (EPIC-26) Quality of Life Survey, as described by Wei et al. (2000) (Einstein et al., 2019), alongside the Medical Outcomes Study SF-12v2, as examined by Frieling, Davis, and Chiang (2013), were utilized to assess the QoL and treatments in PCS. An analysis of the inter-scale correlation between the EPIC and SF-12 highlighted that co-administering the EPIC-26 with the SF-12v2 facilitates an efficient assessment of QoL. This study encompassed seven continuous QoL variables: urinary incontinence, urinary obstructive symptoms, bowel, sexual, and hormonal symptoms (from EPIC-26), along with physical and mental health conditions (from SF-12v2). All variables were scored to a scale ranging from 0 to 100 for standardized assessment with higher score indicating better QoL. Categorical treatments variables are also included here.

## Care service utilizations

Care service utilization was measured using a self-developed scale. This was developed drawing on three principal sources: the methodological framework of a preceding study by Cockle-Hearne et al. (2013) which relied on evidence from clinical specialists, the findings from a comprehensive systematic review conducted by (King et al., 2015)and consultations with key stakeholders from the Prostate Cancer Foundation NZ and the Cancer Society NZ. The scale comprises 19 items across three categories: advice or support received from clinical professionals (9 items), non-governmental organizations (7 items), and other support sources (3 items). Respondents were asked to indicate whether they had received each type of support, with response options of "YES scores 1" or "NO scores 0." The sum of the total score indicates the extent of using care service with higher scores indicate better service utilizations and it was standardized ranging from 0 to 100. The scale demonstrated a Cronbach’s alpha of 0.83, indicating a high level of internal consistency and confirming the reliability of the items in measuring the intended construct.

## Control variables

Participants' demographic details and medical conditions were incorporated as control variables in the analysis to adjust for potential confounding factors. Control variables included socio-demographic characteristics such as relationship, education, marital status, household income, employment, smoking status, and medical conditions. These were summarized as categorical variables and one of the categories was used as reference for comparison.

# References

Boyes, A., Girgis, A., & Lecathelinais, C. (2009). Brief assessment of adult cancer patients' perceived needs: development and validation of the 34-item Supportive Care Needs Survey (SCNS-SF34). *Journal of Evaluation in Clinical Practice*, *15*(4), 602-606. https://doi.org/10.1111/j.1365-2753.2008.01057.x

Cockle-Hearne, J., Charnay-Sonnek, F., Denis, L., Fairbanks, H., Kelly, D., Kav, S., . . . Jensen, B. (2013). The impact of supportive nursing care on the needs of men with prostate cancer: a study across seven European countries. *British Journal of Cancer*, *109*(8), 2121-2130.

Einstein, D. J., Patil, D., Chipman, J., Regan, M. M., Davis, K., Crociani, C. M., . . . Chang, P. (2019). Expanded Prostate Cancer Index Composite-26 (EPIC-26) online: validation of an internet-based instrument for assessment of health-related quality of life after treatment for localized prostate Cancer. *Urology*, *127*, 53-60.

Garvey, G., Beesley, V. L., Janda, M., Jacka, C., Green, A. C., O’Rourke, P., & Valery, P. C. (2012). The development of a supportive care needs assessment tool for Indigenous people with cancer. *BMC Cancer*, *12*(1), 1-10.

Hoeta, T. J., Baxter, G. D., Bryant, K. A. P., & Mani, R. (2020). Māori pain experiences and culturally valid pain assessment tools for Māori: A systematic narrative review. *New Zealand Journal of Physiotherapy*, *48*(1), 37–50-37–50.

Ream, E., Quennell, A., Fincham, L., Faithfull, S., Khoo, V., Wilson-Barnett, J., & Richardson, A. (2008). Supportive care needs of men living with prostate cancer in England: a survey. *British Journal of Cancer*, *98*(12), 1903-1909.

King, A. J. L., Evans, M., Moore, T. H. M., Paterson, C., Sharp, D., Persad, R., & Huntley, A. L. (2015). Prostate cancer and supportive care: a systematic review and qualitative synthesis of men’s experiences and unmet needs. *European Journal of Cancer Care*, *24*(5), 618–634. https://doi.org/10.1111/ECC.12286

# 
