# Supplementary material for: Quality of life and supportive care needs in prostate cancer: the impact of treatment received and care service utilization among Māori and non-Māori patients in New Zealand
Source: Support Care Cancer. 2025 May 20;33(6):483. doi: 10.1007/s00520-025-09521-7 (PMC12092496; doi:10.1007/s00520-025-09521-7)
Supplement: Supplementary file 2 — Supplementary Material 2 (PDF 904 KB) [file 520_2025_9521_MOESM2_ESM.pdf]

University of Otago
Te Whare Wānanga o Otāgo
New Zealand
Sapere Aude

Department of Anatomy
Te Tari Kikokiko

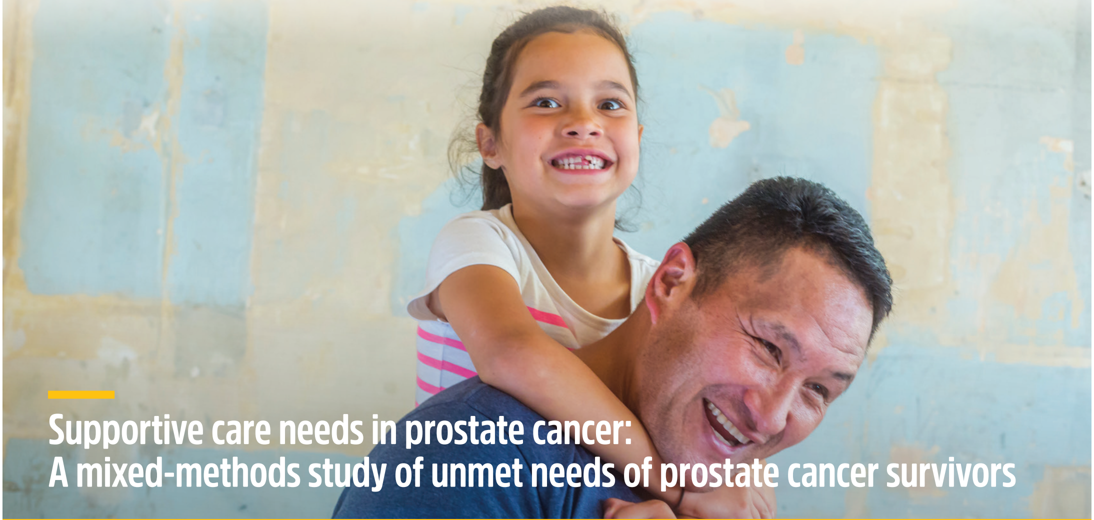

Supportive care needs in prostate cancer: A mixed-methods study of unmet needs of prostate cancer survivors

UNIVERSITY of OTAGO
Te Whare Wānanga o Otāgo
NEW ZEALAND
SAPERE AUDE

Centre for Men's Health

We invite you to take part in the "Supportive care needs in prostate cancer: A mixed-methods study of unmet needs of prostate cancer survivors" survey.

We would like to hear from you about how much support you receive and any ongoing supportive care needs following prostate cancer diagnosis. Your feedback is highly appreciated and will contribute to improving your health and wellbeing. All responses are confidential. This survey should take 15–20 minutes to complete.

To participate in this survey, please:

1) Go to Otago.ac.nz/suppro

2) Click "Complete the survey" and please make sure you complete the questionnaire.

Alternatively, you can scan the QR code to complete the survey.

If you would like a paper copy of the questionnaire or more information:

Email hui.xiao@otago.ac.nz | **Tel** 022 108 9198

SUP0000

M00970-HSCI-HM-20220621

UNIVERSITY of OTAGO
Te Whare Wānanga o Otāgo
NEW ZEALAND

Department of Anatomy
Te Tari Kikokiko

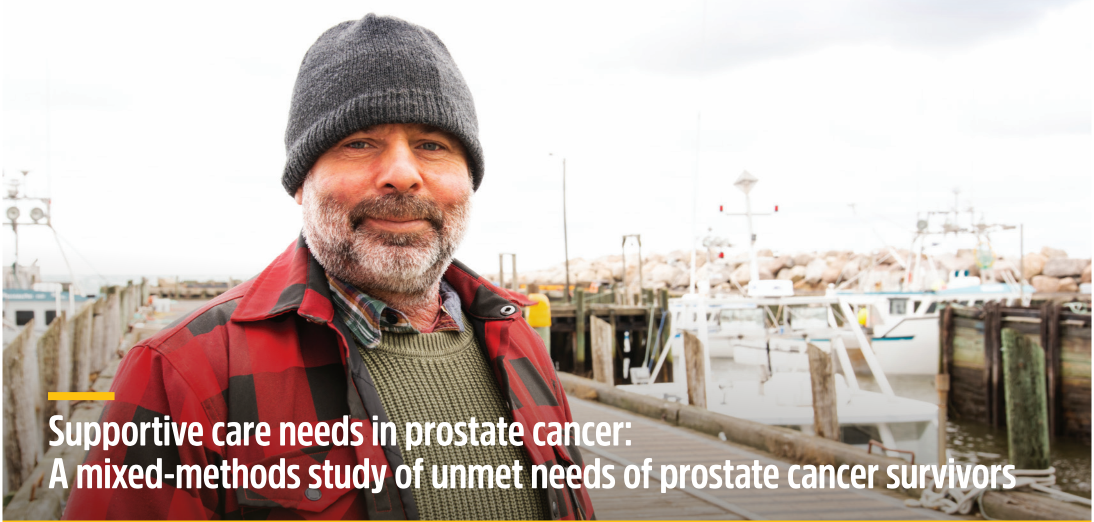

Supportive care needs in prostate cancer: A mixed-methods study of unmet needs of prostate cancer survivors

UNIVERSITY of OTAGO
Te Whare Wānanga o Otāgo
NEW ZEALAND
SAPERE AUDE

Centre for Men's Health

We invite you to take part in the "Supportive care needs in prostate cancer: A mixed-methods study of unmet needs of prostate cancer survivors" survey.

We would like to hear from you about how much support you receive and any ongoing supportive care needs following prostate cancer diagnosis. Your feedback is highly appreciated and will contribute to improving your health and wellbeing. All responses are confidential. This survey should take 15–20 minutes to complete.

To participate in this survey, please:

1) Go to Otago.ac.nz/suppro

2) Click "Complete the survey" and please make sure you complete the questionnaire.

Alternatively, you can scan the QR code to complete the survey.

If you would like a paper copy of the questionnaire or more information:

Email hui.xiao@otago.ac.nz | **Tel** 022 108 9198

SUP0000

M00970-HSCI-HM-20220621
